# Endometrial Microbiome Profiles in Women Evaluated for Infertility or Recurrent Miscarriage: A Single-Center Descriptive Study

**DOI:** 10.3390/diagnostics16121920

**Published:** 2026-06-21

**Authors:** Argyro Papadopoulou, Sofoklis Stavros, Anastasios Potiris, Panagiota Tsoplou, Kyriaki Dioikitopoulou, Vasiliki Plastourgou, Christodoulos Papanikopoulos, Georgios Tournas, Efthalia Moustakli, Athanasios Zikopoulos, Sofia Anysiadou, Anastasia Maria Daskalaki, Panagiotis Antsaklis, Georgios Daskalakis, Ekaterini Domali

**Affiliations:** 1 First Department of Obstetrics and Gynecology, Alexandra Hospital, Medical School, National and Kapodistrian University of Athens, 11528 Athens, Greece; sofanysi@yahoo.gr (S.A.); anastasia.daskalaki00@gmail.com (A.M.D.); panosant@gmail.com (P.A.); gdaskalakis@yahoo.com (G.D.); kdomali@yahoo.fr (E.D.); 2 Third Department of Obstetrics and Gynecology, University General Hospital “ATTIKON”, Medical School, National and Kapodistrian University of Athens, 12462 Athens, Greece; sfstavrou@med.uoa.gr (S.S.); apotiris@med.uoa.gr (A.P.); 3 GeneDiagnosis Genetics Lab, 11525 Athens, Greece; ptsoplou@gmail.com (P.T.); kdioikitopoulou@gmail.com (K.D.); vaswplast@gmail.com (V.P.); 4 HYGEIA IVF Embryogenesis, 15123 Athens, Greece; cpapanikopoulos@yahoo.com; 5 Department of Clinical Therapeutics, Alexandra Hospital, Medical School, National and Kapodistrian University of Athens, 11528 Athens, Greece; tournasg@gmail.com; 6 Department of Nursing, School of Health Sciences, University of Ioannina, 45500 Ioannina, Greece; ef.moustakli@uoi.gr; 7 Obstetrics and Gynecology, Royal Cornwall Hospital, Truro TR1 3LJ, UK; thanzik92@gmail.com

**Keywords:** endometrial microbiome, infertility, *Lactobacillus*, next-generation sequencing, recurrent miscarriage

## Abstract

**Background/Objectives:** The role of the endometrial microbiome in reproductive failure remains incompletely understood. This study aimed to describe the composition of the endometrial microbiome in women evaluated for infertility or recurrent miscarriage. **Methods:** In this single-center descriptive study, endometrial samples were collected from women evaluated for infertility or recurrent miscarriage. Microbiome profiling was performed using 16S rRNA gene next-generation sequencing. Samples were classified as *Lactobacillus*-dominant when *Lactobacillus* spp. accounted for ≥90% of the total bacterial community. Alpha diversity was assessed using the Shannon and Simpson indices, while beta diversity was evaluated using Bray–Curtis dissimilarity, principal coordinates analysis (PCoA), PERMANOVA, and PERMDISP. **Results:** Of the 60 samples, 20 (33.3%) were *Lactobacillus*-dominant and 40 (66.7%) were non-*Lactobacillus*-dominant. Across all samples, Firmicutes was the predominant phylum (76.6%). Non-*Lactobacillus*-dominant samples showed significantly higher alpha diversity than *Lactobacillus*-dominant samples for both the Shannon and Simpson indices (*p* = 1.19 × 10^−6^ and *p* = 1.51 × 10^−6^, respectively), as well as higher observed taxa richness (*p* = 0.000017). PCoA based on Bray–Curtis dissimilarity demonstrated clear separation between microbiome profiles, supported by PERMANOVA (pseudo-F = 13.87, R^2^ = 0.193, *p* = 0.001). PERMDISP showed significantly greater dispersion among non-*Lactobacillus*-dominant samples (F = 566.94, *p* < 0.001). Non-*Lactobacillus*-dominant samples showed greater representation of *Enterococcus* and *Prevotella*. **Conclusions:** In this cohort non-*Lactobacillus*-dominant communities were more frequent with greater diversity, richness, and compositional heterogeneity than *Lactobacillus*-dominant communities. These findings highlight the need for larger, standardized studies with appropriate control populations to clarify their clinical significance.

## 1. Introduction

Worldwide, approximately 17% of reproductive-aged males and females face infertility issues, with the phenomenon being more prevalent in Western-type societies. In about one third of these cases, a routine diagnostic workup fails to identify a specific cause, often leading to feelings of self-doubt and mistrust towards the healthcare system [1,2]. Although guidelines from highly respected scientific bodies, including ESHRE and ASRM, recommend a structured evaluation of infertile individuals, several aspects of reproductive failure remain insufficiently understood. Among these, the potential role of the human microbiome in fertility, implantation, and early pregnancy maintenance has attracted increasing scientific interest, but a consensus regarding its clinical relevance has not yet been reached [3,4,5].

The female reproductive tract microbiome represents a complex and dynamic ecosystem that may influence local immune responses, epithelial barrier function, inflammation, and susceptibility to ascending infection. Historically, the upper reproductive tract, including the uterine cavity, was considered sterile. In 2011, the landmark study by Ravel et al. classified the vaginal microbiome of reproductive-aged women into five community state types according to the dominance or absence of *Lactobacillus* species [6]. This pioneering work inspired others to further investigate uterine microbiome, ultimately demonstrating that the uterine cavity is not sterile as previously believed.

Recent advances in molecular biology and genomics, particularly next-generation sequencing of the 16S rRNA gene, have provided new insights into the composition of the endometrial microbiome and its possible association with reproductive outcomes. *Lactobacillus*-dominant endometrial communities are generally considered more stable and potentially favorable. In the available literature, reduced *Lactobacillus* predominance is commonly described using relative-abundance thresholds, most often defining non-*Lactobacillus*-dominant profiles as those in which *Lactobacillus* spp. account for less than 90% of the bacterial community. Such profiles have been associated in some studies with impaired implantation, lower pregnancy rates, and reduced chances of term live birth in women undergoing assisted reproductive treatment. However, this threshold should be understood as an operational microbiome classification rather than as a validated diagnostic cut-off or evidence of a proven pathological state [7,8,9,10,11]. However, the biological mechanisms underlying these associations remain insufficiently understood. It has been hypothesized that altered microbial communities may contribute to reproductive failure through local inflammation, disruption of endometrial receptivity, changes in cytokine expression, or impaired immune tolerance during the implantation window.

Given this uncertainty over the potential cause-and-effect relationship between the endometrial microbiome and reproductive failure, ESHRE currently recommends microbiome testing primarily for research purposes in selected individuals [5]. Accordingly, our team incorporated endometrial microbiome assessment into the evaluation of women attending our clinic for infertility or recurrent miscarriage, particularly when routine investigations had not identified an alternative explanation. The present study aimed to describe the endometrial microbiome composition in this cohort, classify samples according to *Lactobacillus* dominance, and compare microbial diversity and taxonomic profiles between *Lactobacillus*-dominant and non-*Lactobacillus*-dominant communities.

## 2. Materials and Methods

### 2.1. Ethical Approval and Study Setting

This was a single-center observational study conducted at Alexandra University Hospital in Athens, Greece. The study was approved by the Research Ethics Committee of the National and Kapodistrian University of Athens under protocol number 45203, with approval granted on 2 May 2025. All study procedures were performed in accordance with the ethical principles outlined in the Declaration of Helsinki [12].

Women were recruited among patients attending the outpatient reproductive medicine and gynecology services of Alexandra University Hospital for evaluation of infertility or recurrent miscarriage. Prior to inclusion, all participants received information regarding the purpose of the study, the sampling procedure, the type of data collected, and the potential use of anonymized clinical and microbiome data for scientific and research purposes. Written informed consent was obtained from all participants before endometrial sampling was performed. All data were anonymized before analysis, and each participant was assigned a unique study identifier to ensure confidentiality.

Eligible participants were women of any reproductive age undergoing evaluation for infertility or recurrent miscarriage. Infertility was defined as failure to achieve a clinical pregnancy after at least 12 months of regular unprotected intercourse. Women were included in this group when standard infertility investigations, including semen analysis, assessment of tubal patency, and basic endocrine evaluation, had not identified an alternative explanatory cause. Recurrent miscarriage was defined as the loss of two or more first-trimester pregnancies. Women were considered eligible if they were clinically suitable for endometrial sampling and had provided written informed consent for the use of their anonymized data for research purposes.

### 2.2. Endometrial Sampling Procedure, Sample Handling and Storage

Endometrial sampling was conducted during the mid-luteal phase of the menstrual cycle, corresponding to the expected window of implantation. The procedure was brief, typically lasting 1–2 min and was performed without the need for anesthesia. Following placement of the patient in the lithotomy position, the perineum, vagina, and cervix were thoroughly cleansed using sterile saline to minimize contamination that could compromise NGS analyses, given the low-biomass composition of the uterine microbiome. A sterile speculum was then inserted to allow visualization of the cervix. Endometrial tissue was collected using a sterile Pipelle catheter. Upon advancing the catheter to the uterine fundus, negative pressure was generated by withdrawing the plunger. The device was then gently rotated and retracted while maintaining suction, enabling the acquisition of an adequate endometrial sample.

Immediately after collection, each specimen was labeled with a unique anonymized identifier and transferred into a sterile cryotube pre-filled with nucleic acid stabilization medium. Samples were either stored at 4 °C when processing was expected within a few hours or frozen at −80 °C for long-term preservation. This handling protocol was used to preserve microbial DNA integrity and reduce the risk of degradation before downstream molecular analysis [13].

To ensure a standardized and clinically feasible sampling approach across the cohort, a single endometrial sample was collected from each participant. Although repeated sampling could provide information on temporal microbiome variability, it was not considered appropriate in the present clinical setting because repeated endometrial biopsy is invasive, increases participant discomfort and burden, and incurs additional cost. Therefore, a single mid-luteal sample was considered the most pragmatic approach for this observational study.

### 2.3. Microbiome Analysis by 16S rRNA Gene Next-Generation Sequencing

Total bacterial DNA was extracted from endometrial biopsy specimens using a CE IVD certified DNA extraction kit according to the manufacturer’s instructions. DNA quality and quantity were assessed prior to sequencing, using the Qubit™ 1X dsDNA HS Assay Kit (Invitrogen™, Thermo Fisher Scientific, Waltham, MA, USA) (High Sensitivity).

Bacterial 16S rRNA gene libraries were generated using the Ion 16S Metagenomics Kit (Thermo Fisher Scientific, Waltham, MA, USA), which amplifies seven hypervariable regions (V2, V3, V4, V6, V7, V8, and V9) of the 16S rRNA gene to improve taxonomic resolution at the genus and species level. Amplicons were barcoded with Ion Xpress™ adapters(Thermo Fisher Scientific, Waltham, MA, USA), purified (Vahts DNA Clean Beads), quantified, and pooled at equimolar concentrations [14].

Template preparation and chip loading were performed on the Ion Chef Instrument (Thermo Fisher Scientific), and sequencing was carried out on the Ion GeneStudio S5 System (Thermo Fisher Scientific) using semiconductor next-generation sequencing [15].

Primary sequencing data processing was performed through the Ion Torrent/Torrent Suite workflow, including base calling, quality control, and demultiplexing. Downstream taxonomic profiling was performed using the Ion Reporter™ Metagenomics Workflow with MicroSEQ™ ID and Greengenes databases (Thermo Fisher Scientific). The Ion 16S Metagenomics Kit amplifies seven 16S rRNA hypervariable regions (V2, V3, V4, V6, V7, V8, and V9). These regions were evaluated within the Ion Reporter™/MicroSEQ ID workflow to generate a single taxonomic profile for each sample. The MicroSEQ™ ID 16S rRNA database was used to support high-confidence species-level identification, while the Greengenes database supported broader taxonomic assignment, including genus-level classification. Taxonomic calls were therefore based on the platform-generated proprietary classification output rather than on an independent ASV- or OTU-based bioinformatic pipeline. Taxonomic profiles were analyzed using relative-abundance data generated after quality filtering and chimera removal. Relative abundances of bacterial taxa were calculated at the phylum, family, genus, and species levels when assignable. Unclassified or non-assignable reads were retained as unclassified/other taxa where applicable and were not manually reassigned [7,16].

According to the laboratory workflow, positive-control samples were not included in the sequencing analysis of the present endometrial biopsy specimens. Positive-control samples had been used previously during the initial method-validation stage in early 2023, together with clinical samples. For the present analysis, the laboratory applied internal contaminant-exclusion guidelines. Microorganisms belonging to taxa previously recorded or reported as possible laboratory contaminants, including Sphingomonadaceae, Methylobacteriaceae, Rhodobacteraceae, Xanthomonadaceae, and Rhizobium, were excluded from the analysis and were therefore not included in the final taxonomic profiles. Endometrial biopsy samples with satisfactory DNA concentration but low sequencing output, defined as fewer than 30,000 reads in Ion Reporter™, were not evaluated. Analyses were therefore based on samples that passed the laboratory sequencing-quality criteria. No rarefaction was performed; diversity metrics and taxonomic summaries were calculated from the final post-filtering relative-abundance taxonomic profiles.

Samples were classified as *Lactobacillus*-dominant when *Lactobacillus* spp. accounted for ≥90% of the bacterial community. Samples with *Lactobacillus* relative abundance < 90% were classified as non-*Lactobacillus*-dominant. This threshold was used as an operational microbiome classification criterion and was not intended to define a validated diagnostic cut-off or a proven pathological state.

For transparency, per-sample sequencing and diversity information is provided in Supplementary Table S1, including available read counts, *Lactobacillus* relative abundance, classification status, observed richness, Shannon index, Simpson index, and dominant genera.

### 2.4. Post-Test Clinical Management

Post-test clinical management was not part of the study objectives and was not evaluated as an intervention. When a non-*Lactobacillus*-dominant endometrial microbiome profile was identified, subsequent management was individualized by the treating clinician according to the patient’s microbiological findings, clinical context, reproductive plan, and antimicrobial susceptibility testing when available. Antibiotic or probiotic use after microbiome assessment was not standardized within the study protocol, and no systematic pre-/post-treatment microbiome assessment was performed.

### 2.5. Data Collection and Analysis

Baseline demographic, lifestyle, clinical, reproductive, and sociodemographic characteristics were recorded for all participants at the time of enrolment. Data were collected from clinical records and participant-reported information during the initial evaluation and were entered into an anonymized study database using the unique identifier assigned to each participant. These variables were selected to describe the study population and to capture factors that may be relevant to reproductive health, infertility, pregnancy loss, and microbiome composition.

Demographic and anthropometric variables included age, height, and weight. Body mass index (BMI) was calculated from recorded height and weight and expressed as kg/m^2^. Lifestyle characteristics included smoking status, alcohol consumption, and caffeine intake. Smoking status was classified as non-smoker, current smoker, occasional smoker, or previous smoker. Alcohol and caffeine consumption were recorded according to the participant’s usual intake. Relevant medical history was also documented, including thyroid disease, diabetes, and other comorbidities, as these conditions may be associated with reproductive outcomes or may influence clinical management.

Reproductive and gynecological history was collected in detail. Recorded variables included gravidity, parity, history of miscarriage, history of ectopic pregnancy, history of termination of pregnancy, previous gynecological surgery, and age at menarche. These data were included to provide a comprehensive clinical profile of the cohort and to allow description of previous reproductive outcomes. The primary indication for evaluation was classified as infertility or recurrent miscarriage, according to the eligibility definitions used in the study.

Sociodemographic characteristics such as nationality, race, religion, education level, and socioeconomic status were additionally documented. For the purposes of this study, socioeconomic status (SES) was classified into three categories based on annual income, using cut-offs derived from the ELSTAT EU-SILC 2024 survey (income year 2023): low SES (≤€9513/year), middle SES (€9514–€12,150/year), and high SES (≥€12,151/year) [17]. Education status was classified according to the International Standard Classification of Education (ISCED) as low education (ISCED 0–2), medium education (ISCED 3–4), and high education (ISCED 5–8) [18].

Continuous variables were summarized as mean ± standard deviation (SD) when approximately normally distributed, or as median and interquartile range (IQR) when skewed. Categorical variables were presented as frequencies and percentages.

### 2.6. Microbial Diversity Metrics

Microbial diversity within each endometrial sample was assessed using alpha diversity metrics, specifically the Shannon and Simpson indices. The Shannon index accounts for both the number of different taxa (richness) and the relative abundance of each taxon (evenness). Higher Shannon values indicate a more diverse and evenly distributed microbiome, whereas lower values reflect dominance by one or a few taxa. The Simpson index was reported as 1−D. This metric is derived from the probability that two randomly selected individuals from a given sample belong to the same taxon; when expressed as 1 − D, higher values indicate greater diversity, whereas lower values indicate dominance by one or a few taxa.

The Mann–Whitney U test was used to compare alpha diversity indices between microbiome groups.

To further describe the taxonomic composition of non-*Lactobacillus*-dominant communities, an exploratory genus-level analysis was performed. Key non-*Lactobacillus* genera were selected based on their mean relative abundance in non-*Lactobacillus*-dominant samples, excluding *Lactobacillus* and unclassified/other taxa. The relative abundance of these selected genera was then compared between *Lactobacillus*-dominant and non-*Lactobacillus*-dominant samples using the Mann–Whitney U test. Given the data-driven and exploratory nature of this analysis, *p*-values were interpreted descriptively and no adjustment for multiple comparisons was applied [19,20].

Between-sample (beta) diversity was assessed to characterize differences in overall microbiome composition across study participants. Beta diversity was quantified using Bray–Curtis dissimilarity, and the resulting distance matrix was visualized by principal coordinates analysis (PCoA). Permutational multivariate analysis of variance (PERMANOVA) was applied to test for global differences in microbiome composition between *Lactobacillus*-dominant vs. non-*Lactobacillus*-dominant microbiomes. Because PERMANOVA may be influenced by differences in within-group dispersion, permutational analysis of multivariate dispersion (PERMDISP) was additionally performed using Bray–Curtis dissimilarity to assess whether multivariate dispersion differed between microbiome groups. Accordingly, beta-diversity findings were interpreted as evidence of overall compositional differences between groups, potentially reflecting both differences in group centroids and differences in within-group dispersion [21,22].

In addition, exploratory analyses were conducted to assess whether microbiome profile was associated with the primary indication for evaluation (infertility vs. recurrent miscarriage) and with a history of at least one previous miscarriage (yes vs. no). Associations between categorical variables were evaluated using Fisher’s exact test. All tests were two-sided, and *p* < 0.05 was considered statistically significant.

To address the threshold-dependent nature of the *Lactobacillus*-dominant versus non-*Lactobacillus*-dominant comparison, exploratory correlation analyses were performed using *Lactobacillus* relative abundance as a continuous variable. Spearman’s rank correlation was used to assess associations between *Lactobacillus* relative abundance and observed richness, Shannon index, and Simpson index (1 − D).

Statistical analyses reported in this study were conducted using Stata Statistical Software: Release 17 (StataCorp LLC, College Station, TX, USA).

## 3. Results

From 3 May to 31 December 2025, a total of 60 women were recruited and included in the final analysis. No participants were excluded after recruitment. The mean age of the study population was 37.1 years, with a standard deviation of 6.9 years, indicating that the cohort mainly consisted of women in the later reproductive age range. The mean age at menarche was 12.2 ± 1.3 years, while the mean body mass index (BMI) was 23.3 ± 2.6 kg/m^2^, corresponding overall to a normal-weight population. The main indication for evaluation was infertility, which accounted for 48/60 cases (80.0%). The remaining 12/60 women (20.0%) were evaluated for recurrent miscarriage. Regarding reproductive history, 21/60 participants (35.0%) had experienced at least one previous pregnancy, whereas only 5/60 women (8.3%) had a history of at least one live birth. A history of miscarriage was reported by 19/60 participants (31.7%), while previous termination of pregnancy and ectopic pregnancy were reported by 7/60 (11.7%) and 6/60 women (10.0%), respectively. The cohort was relatively homogeneous in terms of demographic and sociodemographic characteristics. Most participants were Greek (96.7%), of Caucasian/White ethnicity (98.3%), and Christian Orthodox (98.3%). Most women had high educational attainment, with 41/60 participants (68.3%) classified as having high education according to ISCED criteria. The majority also belonged to the high socioeconomic status category according to the predefined income-based classification. Detailed baseline demographic, clinical, lifestyle, reproductive, and sociodemographic characteristics are presented in Table 1.

Baseline characteristics stratified by endometrial microbiome profile are presented in Supplementary Table S2. Overall, *Lactobacillus*-dominant and non-*Lactobacillus*-dominant groups were broadly comparable with respect to age, BMI, current/occasional smoking, primary indication for evaluation, miscarriage history, thyroid disease, and diabetes. No statistically significant differences were identified between the two groups for these baseline characteristics.

Endometrial microbiome profiling identified two distinct microbiome patterns across the 60 analyzed samples. Overall, 20/60 samples (33.3%) were classified as *Lactobacillus*-dominant, whereas 40/60 samples (66.7%) were classified as non-*Lactobacillus*-dominant. Therefore, non-*Lactobacillus*-dominant communities represented the most frequent microbiome profile in this cohort.

**Figure 1:**
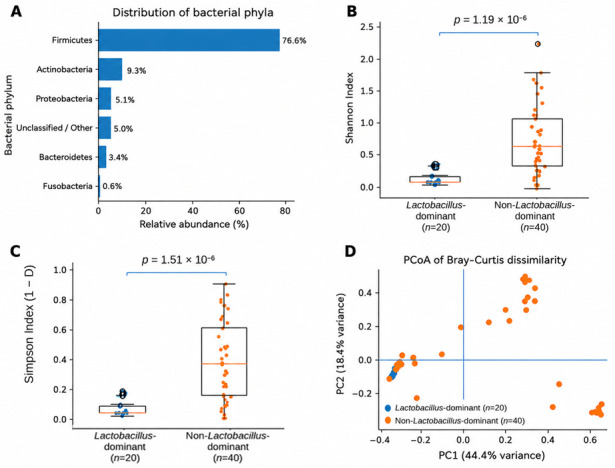
Composition and diversity of the endometrial microbiome in 60 samples stratified by microbiome profile. (**A**) Mean relative abundance of the major bacterial phyla across all samples. (**B**) Shannon index according to microbiome profile, showing significantly higher alpha diversity in non-*Lactobacillus*-dominant samples compared with *Lactobacillus*-dominant samples. (**C**) Simpson index (1 − D) according to microbiome profile, also showing significantly higher alpha diversity in non-*Lactobacillus*-dominant samples compared with *Lactobacillus*-dominant samples. In panels (**B**,**C**), individual coloured dots represent individual samples; boxes indicate the interquartile range, the horizontal line within each box indicates the median, whiskers indicate the data range according to the boxplot convention, and black circles indicate outliers. (**D**) Principal coordinates analysis (PCoA) based on Bray–Curtis dissimilarity, showing separation between *Lactobacillus*-dominant and non-*Lactobacillus*-dominant samples. The beta-diversity difference was supported by PERMANOVA (pseudo-F = 13.87, R^2^ = 0.193, *p* = 0.001). PERMDISP analysis is presented separately in Supplementary Figure S2.

At the phylum level, Firmicutes was the predominant bacterial phylum, accounting for 76.6% of total relative abundance across all samples. This was followed by Actinobacteria (9.3%), Proteobacteria (5.1%), unclassified/other taxa (5.0%), Bacteroidetes (3.4%), and Fusobacteria (0.6%). Overall, the phylum-level distribution showed that although Firmicutes represented the dominant component of the endometrial microbiome, several additional bacterial phyla were also detected at lower relative abundance (Figure 1).

**Figure 2:**
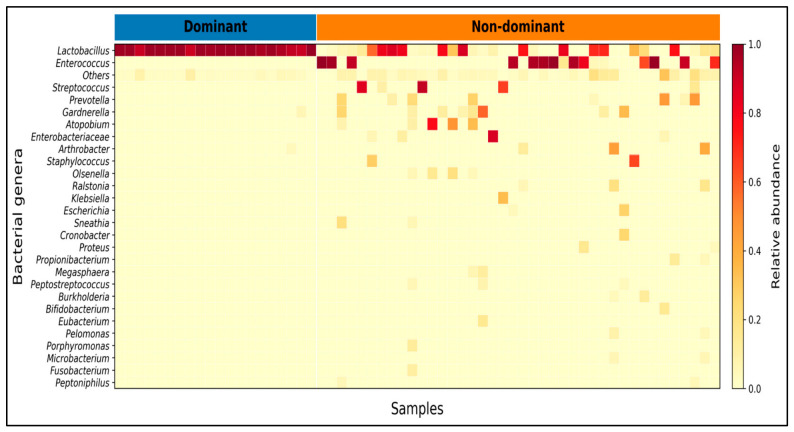
Genus-level heatmap of endometrial microbiome composition according to microbiome profile. Heatmap showing genus-level relative abundance across endometrial samples classified as *Lactobacillus*-dominant or non-*Lactobacillus*-dominant. Samples are grouped by microbiome profile, with *Lactobacillus*-dominant samples shown first and non-*Lactobacillus*-dominant samples shown subsequently. The heatmap illustrates the predominance of *Lactobacillus* in the *Lactobacillus*-dominant group and the broader taxonomic heterogeneity observed among non-*Lactobacillus*-dominant samples. Relative abundance is displayed on a 0–1 scale.

Genus-level heatmap analysis demonstrated clear compositional differences between *Lactobacillus*-dominant and non-*Lactobacillus*-dominant samples. Non-*Lactobacillus*-dominant samples showed a more heterogeneous microbial profile, with increased relative abundance of several non-*Lactobacillus* genera, including *Enterococcus*, *Streptococcus*, *Prevotella*, *Gardnerella*, and *Atopobium* (Figure 2). This pattern suggested broader taxonomic variability among non-*Lactobacillus*-dominant communities.

Alpha diversity differed significantly between the two microbiome groups. *Lactobacillus*-dominant samples had significantly lower within-sample diversity than non-*Lactobacillus*-dominant samples, both for the Shannon index and the Simpson index (1 − D) (*p* = 1.19 × 10^−6^ and *p* = 1.51 × 10^−6^, respectively) (Figure 1). Observed taxa richness was also significantly higher in non-*Lactobacillus*-dominant samples compared with *Lactobacillus*-dominant samples (*p* = 0.000017), further supporting the presence of greater within-sample microbial diversity in the non-*Lactobacillus*-dominant group (Supplementary Figure S1).

Because the comparison between *Lactobacillus*-dominant and non-*Lactobacillus*-dominant groups is inherently threshold-dependent, we additionally examined *Lactobacillus* relative abundance as a continuous variable. *Lactobacillus* relative abundance was inversely correlated with the observed richness (Spearman’s ρ = −0.380, *p* = 0.0028), Shannon index (ρ = −0.441, *p* = 0.00043), and Simpson index (1 − D) (ρ = −0.444, *p* = 0.00038). These findings support a continuous inverse relationship between *Lactobacillus* predominance and microbial diversity, while remaining descriptive in nature.

In addition to differences in alpha diversity, beta-diversity analysis also demonstrated distinct microbial community structures between the two groups. PCoA based on Bray–Curtis dissimilarity showed clear separation between *Lactobacillus*-dominant and non-*Lactobacillus*-dominant samples, with PC1 and PC2 explaining 44.4% and 18.4% of the variance, respectively. This separation was supported by PERMANOVA, which confirmed a statistically significant difference in overall microbial community composition between the two microbiome profiles (pseudo-F = 13.87, R^2^ = 0.193, *p* = 0.001) (Figure 1). Because PERMANOVA may be influenced by differences in within-group dispersion, PERMDISP analysis was performed based on Bray–Curtis dissimilarity. PERMDISP showed significantly greater multivariate dispersion among non-*Lactobacillus*-dominant samples compared with *Lactobacillus*-dominant samples (F = 566.94, *p* < 0.001; Supplementary Figure S2), supporting greater compositional heterogeneity in the non-*Lactobacillus*-dominant group. Therefore, the PERMANOVA result should be interpreted as reflecting overall compositional differences that may include both differences in group centroids and differences in within-group dispersion. Taken together, these findings indicate that the two groups differed not only in the relative abundance of *Lactobacillus*, but also in their broader microbial community composition and within-group heterogeneity.

**Figure 3:**
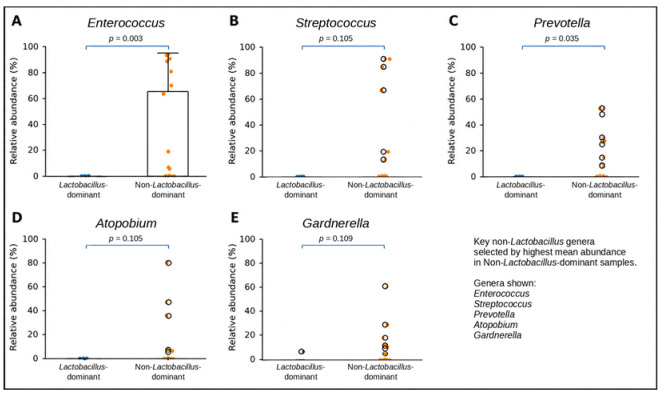
Relative abundance of key non-*Lactobacillus* genera according to microbiome profile. Boxplots show the distribution of genus-level relative abundance (%) in *Lactobacillus*-dominant and non-*Lactobacillus*-dominant endometrial samples. The displayed genera were selected based on the highest mean relative abundance among non-*Lactobacillus*-dominant samples, excluding *Lactobacillus* and unclassified/other taxa. Genus-level comparisons were exploratory; *p*-values are unadjusted and should be interpreted descriptively. (**A**) *Enterococcus*, (**B**) *Streptococcus*, (**C**) *Prevotella*, (**D**) *Atopobium*, and (**E**) *Gardnerella*.

To further characterize the microbial composition of non-*Lactobacillus*-dominant communities, the relative abundance of the main non-*Lactobacillus* genera was compared between non-*Lactobacillus*-dominant and *Lactobacillus*-dominant samples. *Enterococcus* showed markedly higher relative abundance in non-*Lactobacillus*-dominant samples than in *Lactobacillus*-dominant samples (25.1% vs. 0.0%, unadjusted *p* = 0.003). Higher relative abundance was also observed for *Prevotella* (4.7% vs. 0.0%, unadjusted *p* = 0.035). *Streptococcus*, *Atopobium*, and *Gardnerella* were also more abundant in non-*Lactobacillus*-dominant samples, although these exploratory comparisons did not reach conventional statistical significance. Overall, these findings indicate that non-*Lactobacillus*-dominant communities were characterized not only by reduced *Lactobacillus* predominance but also by increased representation of specific alternative genera (Figure 3).

Given the relatively small sample size, additional exploratory analyses were limited to selected clinically relevant variables. Microbiome profile was not significantly associated with the primary indication for evaluation, categorized as infertility or recurrent miscarriage. Specifically, the distribution of *Lactobacillus*-dominant and non-*Lactobacillus*-dominant profiles did not differ significantly between women evaluated for infertility and those evaluated for recurrent miscarriage (Fisher’s exact *p* = 0.511). Likewise, no significant association was identified between microbiome profile and a history of at least one previous miscarriage (yes vs. no; Fisher’s exact *p* = 0.560). These exploratory findings suggest that, within the limits of this sample size, microbiome profile was not clearly associated with these selected clinical characteristics.

## 4. Discussion

In the present study, endometrial microbiome profiling identified two distinct microbial patterns, namely *Lactobacillus*-dominant and non-*Lactobacillus*-dominant communities. The non-*Lactobacillus*-dominant profile was more frequent in our cohort and was characterized by significantly higher alpha diversity, greater observed taxa richness, and significantly different beta diversity compared with *Lactobacillus*-dominant samples. In addition, non-*Lactobacillus*-dominant samples showed increased relative abundance of genera such as *Enterococcus*, *Prevotella*, *Streptococcus*, *Gardnerella*, and *Atopobium*, supporting the presence of a more heterogeneous microbial ecosystem.

The present study adds to the limited evidence on the endometrial microbiome in infertility-related populations by characterizing the endometrial microbiome in a clinically well-characterized cohort of women undergoing evaluation for infertility or recurrent miscarriage. To the best of our knowledge, this may represent one of the first such studies from Greece in this field. This geographical aspect is relevant because microbiome composition may be influenced by population-level factors, including ethnicity, lifestyle, diet, sexual and reproductive history, healthcare practices, antibiotic exposure, and environmental factors. Therefore, data from different populations may contribute to a more complete understanding of the range of endometrial microbiome profiles observed in women with reproductive difficulties.

When interpreting these findings, several methodological strengths and limitations should be considered. The strengths of this study include the use of direct endometrial sampling, detailed baseline phenotyping, and the combined assessment of taxonomic composition together with complementary ecological metrics, including Shannon diversity, Simpson diversity, observed richness, and Bray–Curtis/PCoA/PERMANOVA/PERMDISP. However, several limitations should be acknowledged. This was a single-center study with a relatively small sample size, limiting statistical power and generalizability. In addition, the analysis was based on a single specimen per participant and therefore did not capture temporal microbiome variability. The absence of a control group of fertile women precluded direct comparison with a reference population and limited interpretation of whether the observed microbial profiles were specific to women with reproductive failure. As with all endometrial microbiome research, interpretation is further constrained by the low-biomass nature of uterine samples and the corresponding risk of contamination and methodological variability [23,24]. Although internal contaminant-exclusion procedures were applied, no run-specific sequenced negative controls were processed alongside the clinical samples, and contaminant filtering relied on a predefined laboratory exclusion list rather than on a statistical contaminant-identification approach such as decontam. In addition, raw sequencing reads have not been deposited in a public repository because public raw-read deposition was not covered by the current ethics approval and consent framework, which limits external reproducibility despite the availability of processed supplementary data.

Our findings are broadly consistent with the existing literature, showing that reduced *Lactobacillus* predominance is associated with increased microbial diversity and compositional heterogeneity within the female reproductive tract. The foundational work of Moreno and colleagues established the distinction between *Lactobacillus*-dominated and non-*Lactobacillus*-dominated endometrial profiles and linked non-*Lactobacillus* dominance to poorer reproductive outcomes [7,16,25]. Subsequent studies and recent reviews have generally supported the concept that *Lactobacillus*-dominant communities are associated with a more stable and potentially more favorable reproductive environment, whereas non-*Lactobacillus*-dominant communities are characterized by greater ecological complexity [9,10,26,27,28,29,30,31,32]. In our cohort, this pattern was reflected quantitatively by markedly higher Shannon and Simpson diversity, higher observed taxa richness, and significant separation on beta-diversity analysis in non-*Lactobacillus*-dominant samples. However, these comparisons should be interpreted descriptively, because higher diversity in the non-*Lactobacillus*-dominant group is partly expected from the *Lactobacillus*-based classification threshold. To reduce reliance on the binary cutoff, we additionally examined *Lactobacillus* relative abundance as a continuous variable; this analysis supported an inverse relationship between *Lactobacillus* predominance and observed richness, Shannon diversity, and Simpson diversity.

Recent studies in women with implantation failure, recurrent pregnancy loss, and other forms of reproductive failure point in a similar direction. Contemporary evidence suggests that non-*Lactobacillus* states are not defined by a single alternative genus, but rather by a broader shift toward heterogeneous communities enriched with taxa such as *Enterococcus*, *Streptococcus*, *Gardnerella*, *Prevotella*, and other non-*Lactobacillus* organisms [26,30,31,32]. This is in keeping with our results, in which *Enterococcus* and *Prevotella* showed the clearest enrichment in non-*Lactobacillus*-dominant samples, while *Streptococcus*, *Gardnerella*, and *Atopobium* were also more abundant. At the same time, recent reviews emphasize that no universally accepted pathological endometrial microbiome profile has yet been established, and that differences across studies may partly reflect heterogeneity in sampling methods, sequencing pipelines, patient selection, and reproductive endpoints [23,29].

In the broader context of the available literature, endometrial microbiome profiling has been proposed as a potential research tool for characterizing the endometrial microenvironment in women with unexplained infertility, recurrent miscarriage, repeated implantation failure, or poor reproductive outcomes after assisted reproduction. However, the present study was not designed to evaluate diagnostic accuracy, prognostic value, or treatment efficacy. Therefore, our findings should not be interpreted as establishing the clinical utility of microbiome profiling or supporting its use as a diagnostic test in this setting. At present, endometrial microbiome profiling should be regarded as an investigational approach whose clinical relevance remains to be validated.

In conclusion, this study identified *Lactobacillus*-dominant and non-*Lactobacillus*-dominant endometrial microbiome profiles in a selected cohort of women evaluated for infertility or recurrent miscarriage. Non-*Lactobacillus*-dominant samples were more frequent and showed greater microbial diversity, richness, and compositional heterogeneity. These findings are descriptive and hypothesis-generating. The study does not establish causality, diagnostic accuracy, prognostic value, or treatment efficacy. Larger controlled longitudinal studies with standardized sampling, fertile control groups, harmonized laboratory and bioinformatic workflows, and systematically collected reproductive outcomes are required to clarify the clinical significance of these microbiome patterns. In addition, collaborative microbiome reference initiatives, as illustrated by the Isala vaginal microbiome project, may help establish robust population-level reference profiles and improve biological interpretation across studies [33,34,35,36].

## Figures and Tables

**Table 1:** Baseline demographic, clinical, lifestyle, and sociodemographic characteristics of the study population.

Parameter	Mean (SD) or *n* (%)
**Baseline characteristics**	
Age (years)	37.1 (6.9)
Age at menarche (years)	12.2 (1.3)
Height (m)	1.66 (0.06)
Weight (kg)	64.1 (9.6)
BMI (kg/m^2^)	23.3 (2.6)
**BMI classification**	
Normal weight	44 (73.3)
Overweight	15 (25.0)
Obese	1 (1.7)
**Indication**	
Infertility	48 (80.0)
Recurrent miscarriage	12 (20.0)
**Reproductive history**	
At least one previous pregnancy *	21 (35.0)
At least one live birth **	5 (8.3)
History of miscarriage	19 (31.7)
History of termination of pregnancy	7 (11.7)
History of ectopic pregnancy	6 (10.0)
**Medical history**	
Thyroid disease	14 (23.3)
Diabetes	5 (8.3)
**Smoking status**	
Non-smoker	31 (51.7)
Current smoker	15 (25.0)
Occasional smoker	10 (16.7)
Previous smoking history	4 (6.6)
**Coffee consumption**	
1 coffee/day	31 (51.7)
>1 coffee/day	17 (28.3)
No coffee	12 (20.0)
**Alcohol consumption**	
No alcohol	50 (83.3)
1 drink/day	9 (15.0)
>1 drink/day	1 (1.7)
**Socioeconomic status (SES)**	
High SES (≥€12,151/year)	44 (73.3)
Middle SES (€9514–€12,150/year)	12 (20.0)
Low SES (≤€9513/year)	4 (6.7)
**Education status**	
High (ISCED 5–8)	41 (68.3)
Medium (ISCED 3–4),	14 (23.3)
Low (ISCED 0–2)	5 (8.3)
**Nationality**	
Greek	58 (96.7)
Other	2 (3.3)
**Ethnicity**	
Caucasian/White ethnicity	59 (98.3)
Other	1 (1.7)
**Religion**	
Christian Orthodox	59 (98.3)
Other	1 (1.7)

* Previous pregnancy was defined as any prior clinical pregnancy, irrespective of outcome. Pregnancy outcome categories, including miscarriage, ectopic pregnancy, termination of pregnancy, and live birth, were not mutually exclusive. ** Live birth referred only to pregnancies resulting in a live-born infant.

## Data Availability

Processed data supporting the findings of this study are provided in the Supplementary Materials. Additional de-identified data may be made available by the corresponding author upon reasonable request. Raw sequencing reads have not been deposited in a public repository because public raw-read deposition was not specifically covered by the current ethics approval and consent framework.

## Supplementary Materials

The following supporting information can be downloaded at https://www.mdpi.com/article/10.3390/diagnostics16121920/s1: Figure S1: Observed taxa richness according to microbiome profile. Boxplots show the number of observed taxa per sample in *Lactobacillus*-dominant and non-*Lactobacillus*-dominant endometrial samples. Non-*Lactobacillus*-dominant samples showed significantly higher taxa richness than *Lactobacillus*-dominant samples. Figure S2: Multivariate dispersion according to endometrial microbiome profile. Boxplots show the Bray–Curtis distance of each sample to the centroid of its corresponding microbiome group. Individual points represent endometrial samples, and triangles indicate group means. Non-*Lactobacillus*-dominant samples showed significantly greater distance to the group centroid than *Lactobacillus*-dominant samples, indicating greater within-group compositional heterogeneity (PERMDISP F = 566.94, *p* < 0.001). Table S1: Per-sample sequencing and diversity metrics of the endometrial microbiome. The table presents available per-sample read counts, *Lactobacillus* relative abundance, taxonomic assignment status, microbiome profile, observed richness, Shannon index, Simpson index (1 − D), and dominant genus. *Lactobacillus* relative abundance is reported as a proportion of the total bacterial community. Samples were classified as *Lactobacillus*-dominant when *Lactobacillus* spp. accounted for ≥90% of the bacterial community and as non-*Lactobacillus*-dominant when *Lactobacillus* relative abundance was <90%. Table S2: Baseline characteristics according to endometrial microbiome profile. The table summarizes key demographic and clinical characteristics of women with *Lactobacillus*-dominant and non-*Lactobacillus*-dominant endometrial microbiome profiles, allowing assessment of baseline comparability between the two groups.

## Abbreviations

The following abbreviations are used in this manuscript:

ISCED	International Standard Classification of Education
PCoA	Principal Coordinates Analysis
PERMANOVA	Permutational Multivariate Analysis Of Variance
PERMDISP	Permutational Analysis of Multivariate Dispersion
SES	Socioeconomic status

## References

[1] Eurostat Demography of Europe—2025 Edition. https://ec.europa.eu/eurostat/web/interactive-publications/demography-2025.

[2] Mascarenhas M.N., Flaxman S.R., Boerma T., Vanderpoel S., Stevens G.A. (2012). National, regional, and global trends in infertility prevalence since 1990: A systematic analysis of 277 health surveys. PLoS Med..

[3] Zegers-Hochschild F., Adamson G.D., Dyer S., Racowsky C., de Mouzon J., Sokol R., Rienzi L., Sunde A., Schmidt L., Cooke I.D. (2017). The International Glossary on Infertility and Fertility Care, 2017. Hum. Reprod..

[4] Practice Committee of the American Society for Reproductive Medicine (2021). Fertility evaluation of infertile women: A committee opinion. Fertil. Steril..

[5] Romualdi D., Ata B., Bhattacharya S., Bosch E., Costello M., Gersak K., Homburg R., Mincheva M., Norman R.J., Guideline Group on Unexplained Infertility (2023). Evidence-based guideline: Unexplained infertility. Hum. Reprod..

[6] Ravel J., Gajer P., Abdo Z., Schneider G.M., Koenig S.S., McCulle S.L., Karlebach S., Gorle R., Russell J., Tacket C.O. (2011). Vaginal microbiome of reproductive-age women. Proc. Natl. Acad. Sci. USA.

[7] Moreno I., Garcia-Grau I., Perez-Villaroya D., Gonzalez-Monfort M., Bahceci M., Barrionuevo M.J., Taguchi S., Puente E., Dimattina M., Lim M.W. (2022). Endometrial microbiota composition is associated with reproductive outcome in infertile patients. Microbiome.

[8] Balla B., Illes A., Tobias B., Piko H., Beke A., Sipos M., Lakatos P., Kosa J.P. (2024). The Role of the Vaginal and Endometrial Microbiomes in Infertility and Their Impact on Pregnancy Outcomes in Light of Recent Literature. Int. J. Mol. Sci..

[9] Gao X., Louwers Y.V., Laven J.S.E., Schoenmakers S. (2024). Clinical Relevance of Vaginal and Endometrial Microbiome Investigation in Women with Repeated Implantation Failure and Recurrent Pregnancy Loss. Int. J. Mol. Sci..

[10] Su W., Gong C., Zhong H., Yang H., Chen Y., Wu X., Jin J., Xi H., Zhao J. (2024). Vaginal and endometrial microbiome dysbiosis associated with adverse embryo transfer outcomes. Reprod. Biol. Endocrinol..

[11] Toson B., Simon C., Moreno I. (2022). The Endometrial Microbiome and Its Impact on Human Conception. Int. J. Mol. Sci..

[12] World Medical Association (2013). World Medical Association Declaration of Helsinki: Ethical principles for medical research involving human subjects. JAMA.

[13] Bui B.N., van Hoogenhuijze N., Viveen M., Mol F., Teklenburg G., de Bruin J.P., Besselink D., Brentjens L.S., Mackens S., Rogers M.R.C. (2023). The endometrial microbiota of women with or without a live birth within 12 months after a first failed IVF/ICSI cycle. Sci. Rep..

[14] Klindworth A., Pruesse E., Schweer T., Peplies J., Quast C., Horn M., Glockner F.O. (2013). Evaluation of general 16S ribosomal RNA gene PCR primers for classical and next-generation sequencing-based diversity studies. Nucleic Acids Res..

[15] Human Microbiome Project C. (2012). Structure, function and diversity of the healthy human microbiome. Nature.

[16] Moreno I., Codoner F.M., Vilella F., Valbuena D., Martinez-Blanch J.F., Jimenez-Almazan J., Alonso R., Alama P., Remohi J., Pellicer A. (2016). Evidence that the endometrial microbiota has an effect on implantation success or failure. Am. J. Obstet. Gynecol..

[17] HELLENIC STATISTICAL AUTHORITY INCOME INEQUALITY—2024 Survey on Income and Living Conditions (Income Reference Period: 2023). https://www.statistics.gr/documents/20181/5233dcb8-4ef8-16cd-d04f-0577c1caf548.

[18] OECD, EU, UNESCO-UIS ISCED 2011 Operational Manual Guidelines for Classifying National Education Programmes and Related Qualifications. https://www.oecd.org/content/dam/oecd/en/publications/reports/2015/03/isced-2011-operational-manual_g1g4f697/9789264228368-en.pdf.

[19] Kers J.G., Saccenti E. (2021). The Power of Microbiome Studies: Some Considerations on Which Alpha and Beta Metrics to Use and How to Report Results. Front. Microbiol..

[20] Galloway-Pena J., Hanson B. (2020). Tools for Analysis of the Microbiome. Dig. Dis. Sci..

[21] Tang Z.Z., Chen G., Alekseyenko A.V. (2016). PERMANOVA-S: Association test for microbial community composition that accommodates confounders and multiple distances. Bioinformatics.

[22] Anderson M.J. (2017). Permutational Multivariate Analysis of Variance (PERMANOVA). Wiley StatsRef: Statistics Reference Online.

[23] Lull K., Org E. (2023). Uterine Microbiome: Does the Sampling Technique Matter?. Semin. Reprod. Med..

[24] Molina N.M., Sola-Leyva A., Haahr T., Aghajanova L., Laudanski P., Castilla J.A., Altmae S. (2021). Analysing endometrial microbiome: Methodological considerations and recommendations for good practice. Hum. Reprod..

[25] Moreno I., Franasiak J.M. (2017). Endometrial microbiota-new player in town. Fertil. Steril..

[26] Zhang B., Lin S., Wang S., Chen W., Chen Y., Cao D., Liu Q., Yao Y. (2025). Investigation of the Endometrial Microbiome in Recurrent Pregnancy Loss Individuals: Microbial Imbalance and Network Fragility. Int. J. Womens Health.

[27] Han A.R. (2025). Endometrial microbiome in reproductive failure: The possibility of metagenomic analysis. Clin. Exp. Reprod. Med..

[28] Yao Y., Ye Y., Zheng C. (2025). The Impact of Microbiota-Mediated Immune Regulation on Recurrent Pregnancy Loss and Intervention Strategies. Am. J. Reprod. Immunol..

[29] Karadbhajne P., More A., Dzoagbe H.Y. (2025). The Role of Endometrial Microbiota in Fertility and Reproductive Health: A Narrative Review. Cureus.

[30] Gao H., Xiao J., Liang B., Wang X., Li H., Li G., Wu B. (2025). Main differential endometrial microbiota associated with recurrent implantation failure: A case control study. Front. Endocrinol..

[31] Blazheva S., Pachkova S., Bodurska T., Ivanov P., Blazhev A., Lukanov T., Konova E. (2024). Unlocking the Uterine Code: Microbiota, Immune Cells, and Therapy for Recurrent Reproductive Failure. Microorganisms.

[32] Odendaal J., Black N., Bennett P.R., Brosens J., Quenby S., MacIntyre D.A. (2024). The endometrial microbiota and early pregnancy loss. Hum. Reprod..

[33] Cao C., Bai S., Zhang J., Sun X., Meng A., Chen H. (2022). Understanding recurrent pregnancy loss: Recent advances on its etiology, clinical diagnosis, and management. Med. Rev..

[34] Franasiak J.M., Alecsandru D., Forman E.J., Gemmell L.C., Goldberg J.M., Llarena N., Margolis C., Laven J., Schoenmakers S., Seli E. (2021). A review of the pathophysiology of recurrent implantation failure. Fertil. Steril..

[35] Cakmak H., Taylor H.S. (2011). Implantation failure: Molecular mechanisms and clinical treatment. Hum. Reprod. Update.

[36] Lebeer S., Ahannach S., Gehrmann T., Wittouck S., Eilers T., Oerlemans E., Condori S., Dillen J., Spacova I., Vander Donck L. (2023). A citizen-science-enabled catalogue of the vaginal microbiome and associated factors. Nat. Microbiol..

