# Supplementary material for: Endometrial Microbiome Profiles in Women Evaluated for Infertility or Recurrent Miscarriage: A Single-Center Descriptive Study
Source: Diagnostics (Basel). 2026 Jun 21;16(12):1920. doi: 10.3390/diagnostics16121920 (PMC13297907; doi:10.3390/diagnostics16121920)
Supplement: Supplementary file 1 [file diagnostics-16-01920-s001.zip › diagnostics-4323078-supplementary.pdf]

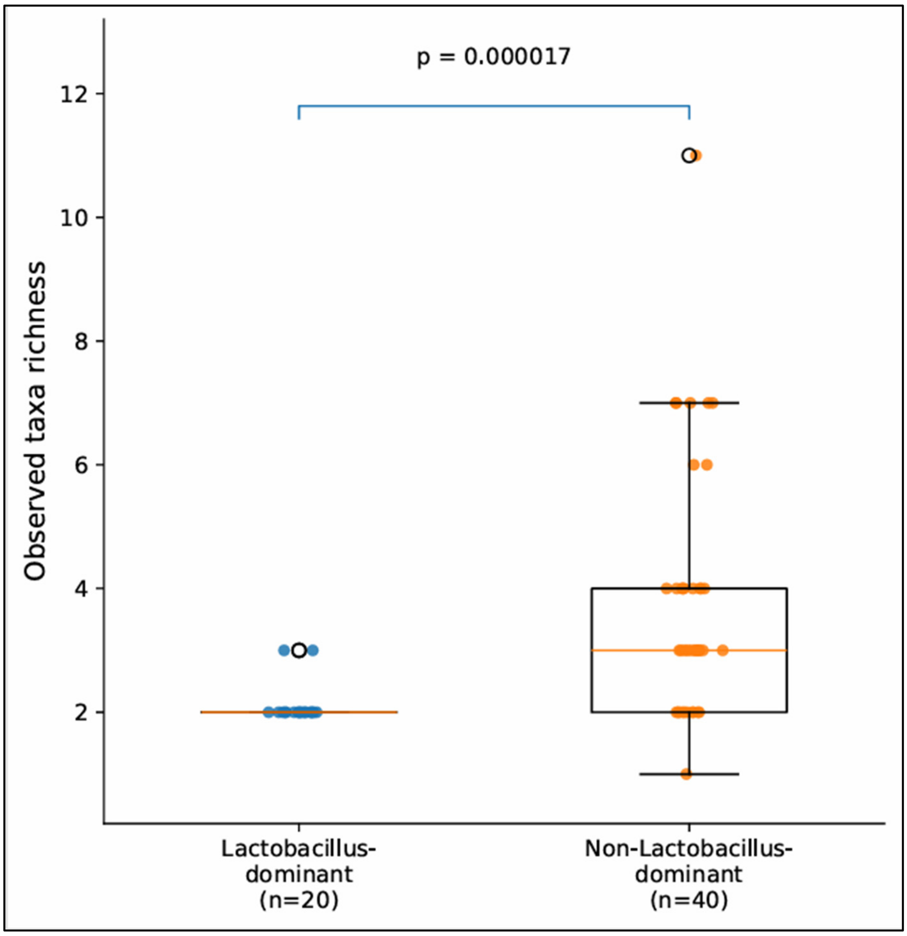

Supplementary Figure S1: Observed taxa richness according to microbiome profile*. Boxplots show the number of observed taxa per sample in Lactobacillus-dominant and non-Lactobacillus-dominant endometrial samples. Non-Lactobacillus-dominant samples showed significantly higher taxa richness than Lactobacillus-dominant samples.*

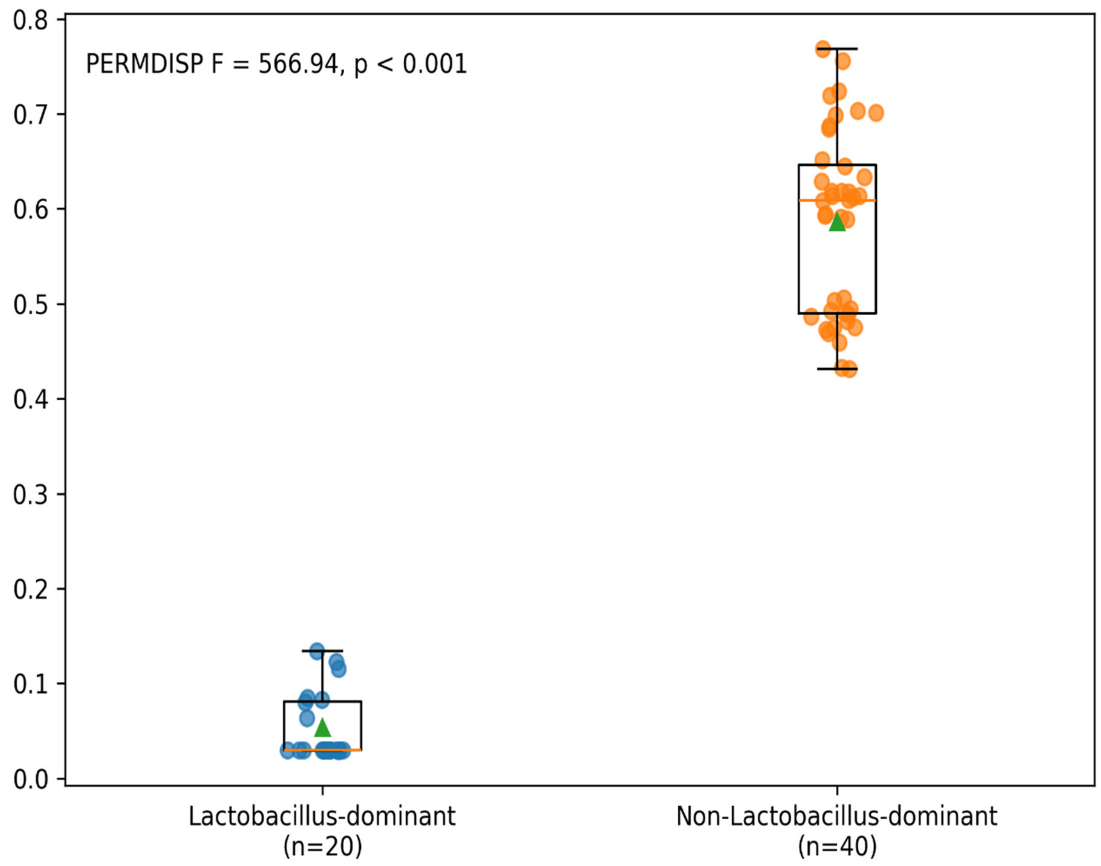

Supplementary Figure S2. Multivariate dispersion according to endometrial microbiome profile. *Boxplots show the Bray–Curtis distance of each sample to the centroid of its corresponding microbiome group. Individual points represent endometrial samples, and triangles indicate group means. Non-Lactobacillus-dominant samples showed significantly greater distance to the group centroid than Lactobacillus-dominant samples, indicating greater within-group compositional heterogeneity (PERMDISP F = 566.94, p < 0.001).*

Code	Lactobacillus relative abundance	Read counts	Classification status	Microbiome profile	Observed richness	Shannon index	Simpson index (1-D)	Dominant genus
1	98%	117350	Classified at genus level	*Lactobacillus*-dominant	2	0,098	0,039	*Lactobacillus*
2	0%	65656	Classified at genus level	non-*Lactobacillus*-dominant	1	0,000	0,000	*Enterococcus*
3	98%	66160	Classified at genus level	*Lactobacillus*-dominant	2	0,098	0,039	*Lactobacillus*
4	3%	65379	Classified at genus level	non-*Lactobacillus*-dominant	2	0,130	0,056	*Enterococcus*
5	91%	75689	Classified at genus level	*Lactobacillus*-dominant	2	0,303	0,164	*Lactobacillus*
6	98%	52908	Classified at genus level	*Lactobacillus*-dominant	2	0,098	0,039	*Lactobacillus*
7	98%	60282	Classified at genus level	*Lactobacillus*-dominant	2	0,098	0,039	*Lactobacillus*
8	98%	212008	Classified at genus level	*Lactobacillus*-dominant	2	0,098	0,039	*Lactobacillus*
9	98%	144149	Classified at genus level	*Lactobacillus*-dominant	2	0,098	0,039	*Lactobacillus*
10	6%	86741	Classified at genus level	non-*Lactobacillus*-dominant	7	1,765	0,806	*Gardnerella*
11	8%	104414	Classified at genus level	non-*Lactobacillus*-dominant	2	0,279	0,147	*Enterococcus*
12	14%	66717	Classified at genus level	non-*Lactobacillus*-dominant	2	0,405	0,241	*Streptococcus*
13	57%	63425	Classified at genus level	non-*Lactobacillus*-dominant	4	1,056	0,582	*Lactobacillus*
14	90%	91015	Classified at genus level	*Lactobacillus*-dominant	2	0,325	0,180	*Lactobacillus*
15	98%	66577	Classified at genus level	*Lactobacillus*-dominant	2	0,098	0,039	*Lactobacillus*
16	82%	56892	Classified at genus level	non-*Lactobacillus*-dominant	3	0,595	0,311	*Lactobacillus*
17	87%	113678	Classified at genus level	non-*Lactobacillus*-dominant	3	0,457	0,232	*Lactobacillus*
18	82%	230041	Classified at genus level	non-*Lactobacillus*-dominant	3	0,592	0,311	*Lactobacillus*
19	3%	93071	Classified at genus level	non-*Lactobacillus*-dominant	11	2,247	0,878	*Prevotella*
20	97%	70737	Classified at genus level	*Lactobacillus*-dominant	2	0,135	0,058	*Lactobacillus*
21	4%	37691	Classified at genus level	non-*Lactobacillus*-dominant	3	0,334	0,150	*Streptococcus*
22	98%	32268	Classified at genus level	*Lactobacillus*-dominant	2	0,098	0,039	*Lactobacillus*
23	98%	79939	Classified at genus level	*Lactobacillus*-dominant	2	0,098	0,039	*Lactobacillus*
24	4%	100454	Classified at genus level	non-*Lactobacillus*-dominant	4	0,712	0,367	*Atopobium*
25	79%	115304	Classified at genus level	non-*Lactobacillus*-dominant	3	0,657	0,353	*Lactobacillus*

26	31%	45685	Classified at genus level	non-*Lactobacillus*-dominant	4	1,118	0,643	*Atopobium*
27	98%	76218	Classified at genus level	*Lactobacillus*-dominant	2	0,098	0,039	*Lactobacillus*
28	88%	83329	Classified at genus level	non-*Lactobacillus*-dominant	3	0,443	0,218	*Lactobacillus*
29	98%	63850	Classified at genus level	*Lactobacillus*-dominant	2	0,098	0,039	*Lactobacillus*
30	96%	52009	Classified at genus level	*Lactobacillus*-dominant	2	0,168	0,077	*Lactobacillus*
31	98%	78638	Classified at genus level	*Lactobacillus*-dominant	2	0,098	0,039	*Lactobacillus*
32	6%	121506	Classified at genus level	non-*Lactobacillus*-dominant	7	1,668	0,772	*Atopobium*
33	95%	88349	Classified at genus level	*Lactobacillus*-dominant	2	0,199	0,095	*Lactobacillus*
34	3%	99580	Classified at genus level	non-*Lactobacillus*-dominant	6	1,291	0,618	*Gardnerella*
35	8%	75483	Classified at genus level	non-*Lactobacillus*-dominant	3	0,443	0,218	*Enterobacter*
36	0%	84723	Classified at genus level	non-*Lactobacillus*-dominant	2	0,641	0,449	*Streptococcus*
37	0%	91549	Classified at genus level	non-*Lactobacillus*-dominant	3	0,265	0,114	*Enterococcus*
38	75%	111482	Classified at genus level	non-*Lactobacillus*-dominant	4	0,819	0,413	*Lactobacillus*
39	5%	74150	Classified at genus level	non-*Lactobacillus*-dominant	2	0,199	0,095	*Enterococcus*
40	0%	112837	Classified at genus level	non-*Lactobacillus*-dominant	2	0,168	0,077	*Enterococcus*
41	0%	53685	Classified at genus level	non-*Lactobacillus*-dominant	2	0,056	0,020	*Enterococcus*
42	83%	89413	Classified at genus level	non-*Lactobacillus*-dominant	3	0,517	0,288	*Lactobacillus*
43	0%	62477	Classified at genus level	non-*Lactobacillus*-dominant	2	0,199	0,095	*Enterococcus*
44	0%	69104	Classified at genus level	non-*Lactobacillus*-dominant	3	0,534	0,302	*Enterococcus*
45	92%	43225	Classified at genus level	*Lactobacillus*-dominant	3	0,334	0,150	*Lactobacillus*
46	70%	57695	Classified at genus level	non-*Lactobacillus*-dominant	4	0,871	0,465	*Lactobacillus*
47	71%	81271	Classified at genus level	non-*Lactobacillus*-dominant	4	0,890	0,463	*Lactobacillus*
48	0%	98526	Classified at genus level	non-*Lactobacillus*-dominant	7	1,591	0,735	*Arthrobacter*
49	0%	105602	Classified at genus level	non-*Lactobacillus*-dominant	6	1,444	0,734	*Gardnerella*

50	36%	98361	Classified at genus level	non-*Lactobacillus*-dominant	2	0,653	0,461	*Staphylococcus*
51	23%	74882	Classified at genus level	non-*Lactobacillus*-dominant	4	0,940	0,533	*Enterococcus*
52	91%	88749	Classified at genus level	*Lactobacillus*-dominant	3	0,360	0,167	*Lactobacillus*
53	99%	63921	Classified at genus level	*Lactobacillus*-dominant	2	0,056	0,020	*Lactobacillus*
54	0%	58487	Classified at genus level	non-*Lactobacillus*-dominant	2	0,056	0,020	*Enterococcus*
55	0%	85692	Classified at genus level	non-*Lactobacillus*-dominant	4	1,193	0,659	*Prevotella*
56	76%	110231	Classified at genus level	non-*Lactobacillus*-dominant	3	0,714	0,393	*Lactobacillus*
57	0%	45217	Classified at genus level	non-*Lactobacillus*-dominant	3	0,350	0,167	*Enterococcus*
58	5%	69527	Classified at genus level	non-*Lactobacillus*-dominant	7	1,529	0,715	*Prevotella*
59	17%	58036	Classified at genus level	non-*Lactobacillus*-dominant	7	1,653	0,757	*Atopobium*
60	18%	96710	Classified at genus level	non-*Lactobacillus*-dominant	4	0,889	0,470	*Enterococcus*

Supplementary Table S1*. *Per-sample sequencing and diversity metrics o the endometrial microbiome. *The table presents available per-sample read counts, Lactobacillus relative abundance, taxonomic assignment status, microbiome profile, observed richness, Shannon index, Simpson index (1–D), and dominant genus. Lactobacillus relative abundance is reported as a proportion of the total bacterial community. Samples were classified as Lactobacillus-dominant when Lactobacillus spp. accounted for ≥90% of the bacterial community and as non-Lactobacillus-dominant when Lactobacillus relative abundance was <90%.*

Characteristic	Lactobacillus-dominant (n=20)	Non-Lactobacillus-dominant (n=40)	p value
Age (years), mean ± SD	37.0 ± 8.0	37.1 ± 6.4	0.923
BMI (kg/m^2), mean ± SD	23.2 ± 1.8	23.3 ± 2.9	0.805
Current/occasional smoking, n (%)	10 (50.0)	19 (47.5)	1.000
Primary indication			0.511
Infertility, n (%)	15 (75.0)	33 (82.5)	
Recurrent miscarriage, n (%)	5 (25.0)	7 (17.5)	
History of ≥1 miscarriage, n (%)	5 (25.0)	14 (35.0)	0.560
Thyroid disease, n (%)	4 (20.0)	10 (25.0)	0.756
Diabetes, n (%)	2 (10.0)	3 (7.5)	1.000
Recent antibiotic/probiotic/hormonal treatment before sampling, n (%)	Not available	Not available	Not applicable

Data are presented as mean ± SD or n (%). P-values were calculated using Welch's t-test for continuous variables and Fisher's exact test for categorical variables. For categorical variables with more than one displayed category, the p-value refers to the overall distribution of the variable. Data on recent antibiotic, probiotic, or hormonal treatment before sampling were not available in the source clinical records.

Supplementary Table S 2. Baseline characteristics according to endometrial microbiome profile. *The table summarizes key demographic and clinical characteristics of women with Lactobacillus-dominant and non-Lactobacillus-dominant endometrial microbiome profiles, allowing assessment of baseline comparability between the two groups.*
